# School environment and physical activity in adolescents from São Paulo city

**DOI:** 10.1038/s41598-021-97671-z

**Published:** 2021-09-13

**Authors:** Gerson Ferrari, Leandro F. M. Rezende, Alex A. Florindo, Grégore I. Mielke, Maria Fernanda Tourinho Peres

**Affiliations:** 1 Escuela de Ciencias de la Actividad Física, el Deporte y la Salud, Universidad de Santiago de Chile (USACH), Las Sophoras 175, Estación Central, Santiago, Chile; 2 Departamento de Medicina Preventiva, Escola Paulista de Medicina, Universidade Federal de São Paulo, São Paulo, Brazil; 3 School of Arts, Sciences and Humanities, University of Sao Paulo, São Paulo, Brazil; 4 Graduate Program in Nutrition in Public Health, Department of Nutrition, School of Public Health, University of Sao Paulo, São Paulo, SP Brazil; 5 School of Human Movement and Nutrition Sciences, The University of Queensland, Brisbane, Australia; 6 Departamento de Medicina Preventiva, Faculdade de Medicina, Universidade de São Paulo, São Paulo, Brazil

**Keywords:** Health care, Risk factors

## Abstract

We examined the association of physical activity (PA) facilities and access to school with total PA and domain-specific PA in adolescents. We enrolled 2610 adolescents (mean: 14.9 years) from Sao Paulo city. The number and presence of sports courts, swimming pools, locker rooms, running/athletics tracks, entrance accessible for student cyclists, bike racks, speed limit signal around the school, and pedestrian crossing were assessed in each school. All participants responded to a questionnaire about frequency and duration of physical education classes, leisure time, and active commuting. Total PA was obtained by adding up all PA domains. Presence of three or more (OR: 1.62; 95% CI: 1.15 to 2.30) sports courts, swimming pool available in usable conditions (OR: 1.45; 95% CI: 1.01 to 2.10), running/athletics tracks (OR: 2.35; 95% CI: 1.07 to 5.18), and bike racks (OR: 1.38; 95% CI: 1.07 to 1.78) were positively associated with total PA. Number of sports courts, swimming pool available in usable conditions, speed limit signals around the school, and pedestrian crossings were positively associated with physical education classes. The bike racks, speed limit signs around the school, and pedestrian crossings were positively associated with active commuting. School environment was associated with increased PA. Our findings should be considered in future epidemiologic studies and for educational and health policy makers.

## Introduction

Several studies have shown the benefits of physical activity for adolescents’ health^1–3^. Physical activity during childhood and adolescence is associated with adiposity status, cardiometabolic biomarkers, mental, psychological, behavioral conduct/pro-social behavior, cognition/academic achievement, and physical fitness in the short, mid- and long-term^2^. Despite these benefits, fewer than 20% of adolescents worldwide and only 29% of Brazilian children aged 11–17 years participate in sufficient physical activity^4^. For adolescents aged 5–17 years, the 2020 World Health Organization Guidelines for physical activity recommends an average of 60 min/day of moderate-to-vigorous physical activity^1^.

Promoting physical activity in adolescents living in megacities is challenging, particularly in low- and middle-income countries which have faced remarkable urbanization in the last decades. By 2030, the world is expected to have 41 megacities, and more than 90% of the future urban population growth will be in low- and middle-income countries^5^. Sao Paulo (Brazil) is the largest megacity in Latin America and one of the largest megacities in the world. With approximately 20.5 million inhabitants^6^, Sao Paulo is characterized by regions with extreme poverty, high occurrence of violence, heavy traffic, air and noise pollution, insecurity spread throughout the city. Moreover, similar to other megacities in low- and middle-income countries, Sao Paulo has faced a fast-paced an unplanned urban growth^7,8^. These changes in the city’s urban environment are likely to impact physical activity^9,10^, as demonstrated by our previous study which showed that only 12.7% of adolescents from Sao Paulo engaged in sufficient physical activity^9^.

Physical activity is determined not solely by individual behavior and choices, but also by sociocultural and environmental factors such as school environments^11,12^. Factors that may affect adolescents’ physical activity during school hours include physical, social, or institutional structures. These factors are in line with ecological models of behavior change in which an environment can influence health behaviors^13,14^.

In addition, monetary aspects may affect the ability of schools to provide opportunities to encourage physical activity among adolescents within the school environment. There is a growing body of research into the influence of the school environment, particularly barriers and enablers, in adolescents’ physical activity^15,16^. A review^16^ and a mixed-method review^15^ demonstrated that schools are more able to enhance adolescents’ physical activity when they emphasize resource provision for physical activity within the school day, create a ‘‘culture’’ of physical activity, train teachers to support a positive climate for physical activity promotion, and ensure extracurricular physical activity opportunities for all adolescents. However, few studies have shown the association between facilities for physical activity in the school and access to school with total and domain-specific physical activity in adolescents living in megacities from low- to middle-income countries^17^.

Given adolescents are likely to spend a substantially amount of daily hours in the school, alteration of physical activity in youth should consider the school environment^9,18,19^. Furthermore, non-availability of, or non-accessibility to activities at school have been reported as main barriers to physical activity involvement among adolescents^20,21^. Therefore, physical activity facilities in schools and access to school are central reasons to be measured in the promotion of physical activity in adolescents^21^.

Studies on adolescents from the high-income countries found significant associations between physical activity facilities and total physical activity^22–24^. However, there are relatively few representative studies about these factors mega cities in low- to middle income countries, particularly in Latin America^25^. Furthermore, to analyze the associations between physical activity facilities and access to school may contribute to the physical activity opportunities of the Latin American adolescents. In this study, we examined the association of physical activity facilities and the access to school with total physical domain-specific physical activity in adolescents from Sao Paulo city, Brazil.

## Methods

### Sao Paulo project for the social development of children and adolescents

*São Paulo para o desenvolvimento social de crianças e adolescentes *(*SP-PROSO—*São Paulo Project for the Social Development of Children and Adolescents) is a cross-sectional study that included a representative sample of 9th grade students from public and private schools in Sao Paulo^9,26^. Data collection was conducted between August and November 2017. The overarching SP-PROSO protocol was approved by the Ethics and Research Committee of the University of Sao Paulo School of Medicine (records no. 1.719.856) and the National Commission for Research Ethics (records no. 2.014.816). Written informed consent was obtained from a parent or legal guardian of all participants from the individual studies. All methods were carried out in accordance with relevant guidelines and regulations. Further details on SP-PROSO have been described elsewhere^9^.

**Figure 1:**
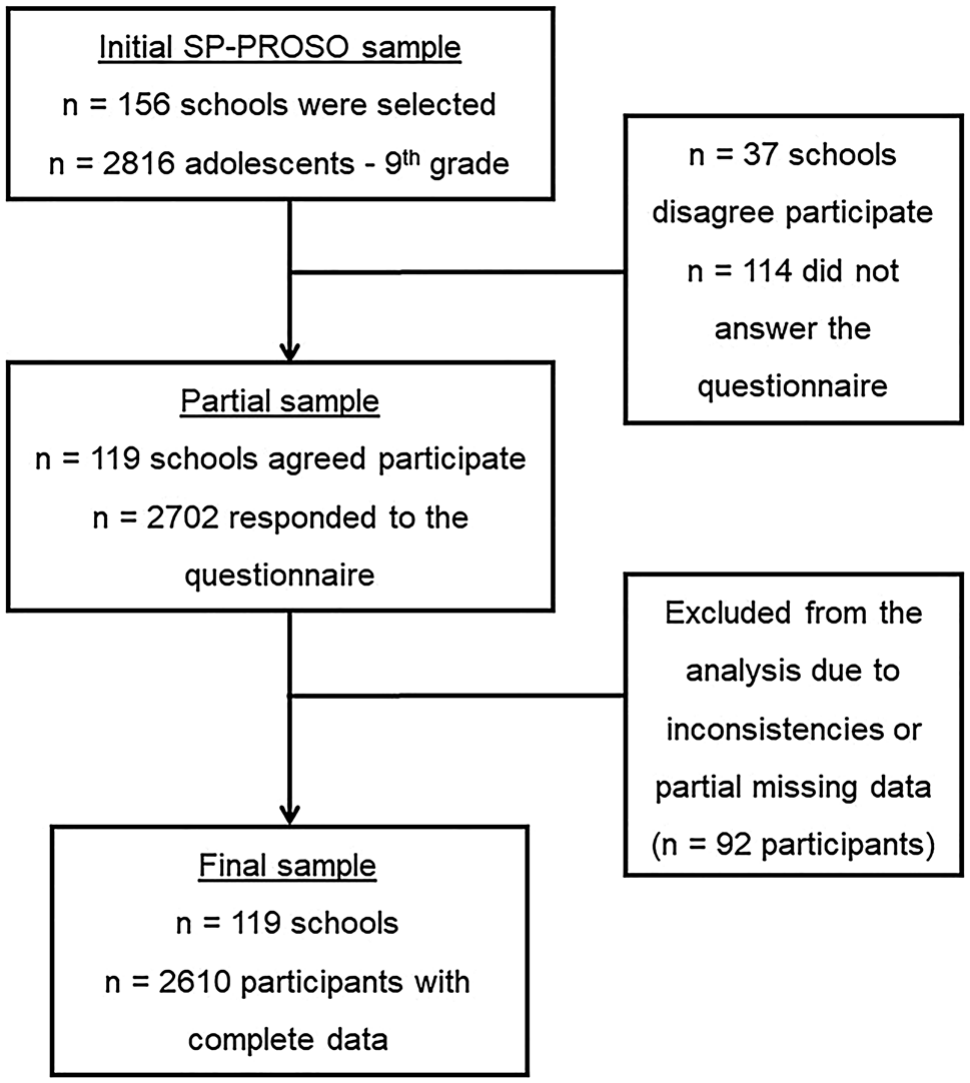
Flow chart of selection of SP-Proso participants.

A multi-stage sampling process was used to select participants of SP-PROSO. According to the school Census conducted in 2015, Sao Paulo had 175,854 ninth-grade students, who were enrolled across 2086 public (state and municipal) and private schools. Initially, 156 schools were randomly selected, of which 119 agreed to participate. Of the 61 private schools drawn, 26 refused to participate and 3 did not respond to our invitation and were excluded. Of the public schools, there was only one loss among the state schools and seven among the municipal schools. In each school, one class was selected and all students were eligible and invited to participle. Of 2816 eligible students, 2702 responded to a questionnaire. Our final analytical sample included 2610 participants with complete information on physical activity, which represented a final response rate of 93% (Fig. 1). Details on participant sampling, recruitment strategies, refused participants, and those who did not respond to our invitation have been published elsewhere^9,26^.

### Physical activity assessment

Participants reported their physical activity levels by completing a self-reported questionnaire. This questionnaire has been used in the National Survey of School Health (2009, and 2019)^27^. The indicators of physical activity from questionnaire showed satisfactory relative validity compared with three 24-h recalls for 300 min/week (sensitivity: 78%; specificity: 69%; accuracy index: 73%) and 150 min/week (sensitivity: 88%; specificity: 49%; accuracy index: 78%) of adolescent´s physical activity^28^.

The participants were instructed to report their weekly frequency and duration of physical activity in the domains of physical education classes, leisure physical activity, and active commuting (walking or cycling) to or from school during the past week.

These questions were asked separately for each domain of physical activity. We calculated total physical activity by adding the minutes per day (min/day) of each domain of physical activity (physical education at school, leisure time physical activity, and active commuting). Total physical activity and leisure time of adolescents was categorized as < 60 min/day or as ≥ 60 min/day, according on the 2020 World Health Organization Recommendation for physical activity^1^. Physical education classes was categorized based on the number of classes offered per week in most Sao Paulo schools^29^ as < 2 or ≥ 2 week. Since only 2.1% of the students met physical activity guidelines thresholds for commuting in our sample and there are no specific guidelines for active commuting, we opt to categorize active commuting on weekly frequency, as none or ≥ 1 week.

### School-environment assessment

The school-environment was assessed using a questionnaire completed by a designated staff member, who observed each physical activity facility and access to school separately. Physical activity facilities and access to school considered were: number of sports courts (none to three or more; categorized as ≤ 1, 2 and ≥ 3), availability of swimming pool (does not have one, it is not in usable conditions, in usable conditions; categorized as none and not fit for use, and in usable conditions), locker room (does not have one, it is not in usable conditions, in usable conditions; categorized as none and not fit for use, and in usable conditions), running/athletics tracks (does not have one, has one), entrance accessible for student cyclists (no, yes), bike rack (no, yes), speed limit signal around the school (no, yes), and pedestrian crossing (no, yes). Type of school (municipal public, state public, and private) also was considered^9,21,30^.

We also generated a composite variable based on the “number of physical activity facilities” at the school by adding the number of physical activity facilities (yes = 1; no = 0) (sports courts, availability of swimming pool, locker room, running/athletics tracks, entrance accessible for student cyclists, bike racks, speed limit signal around the school, and pedestrian crossing. The variable ranged from zero (no facility available) to eight (five facilities evaluated were available and in usable conditions). The number of physical activity facilities was categorized as ≤ 1, 2–4, and ≥ 5.

### Sociodemographic variables

The socioeconomic level was assessed based on mother’s education level (incomplete elementary, incomplete secondary, complete secondary or complete higher) and a series of enquiries that explored the possession of goods and the presence of a maid in the household. These questions have been used in the National School-Based Health Survey (*Pesquisa Nacional de Saúde do Escolar*—PeNSE). The PeNSE survey involved questions about the existence of goods in the home (the home where the adolescents actually lived). This questionnaire and method of evaluating socioeconomic level has been extensively used in Brazilian surveys^9,30^.

The socioeconomic level was designed through principal component analysis (PCA)^30^. PCA was run adding to the model: maternal educational level, type of school, landline, cell phone, computer with internet access in the room, car, and number of bathrooms within the household. Details about the components of the PCA and the equation were published earlier^9,30^. Socioeconomic level was characterized as terciles of the total wealth scores, in which the first tercile (low) is the poorest group and the third tercile (high) is the wealthiest group.

Participants also self-reported their skin color/ethnicity [white, black, mixed, others (yellow, indigenous)] and mother’s employment status (none, part-time, and full-time).

### Statistical analysis

Descriptive statistics included frequency and proportions of adolescent characteristics by total physical activity level (< 60 min/day vs ≥ 60 min/day) and school characteristics. We also calculated the proportion and its respective 95% confidence interval (95% CI) of total and domain-specific physical activity (physical education classes, leisure time physical activity, and active commuting) according to school characteristics.

A multilevel logistic regression model with the number of enrolled students in the school as the first level and type of schools as the second level, were used to estimate the odds ratios (OR) and 95% confidence intervals (95% CI) for the associations of school environment with total physical activity and domain-specific physical activity, adjusted for sex, age (years), skin color/ethnicity, and mother’s employment status. We performed unadjusted and adjusted models including physical activity facilities and access to school separately (number of sports courts, swimming pools, locker rooms, running/athletics tracks, accessible entrance for student cyclists, bike racks, speed limit signal around the school, pedestrian crossing, and number of physical activity facilities) for total physical activity and each domain of physical activity.

Statistical analyses were carried out using SPSS v.26 (SPSS Inc., IBM Corp., Armonk, New York, NY, USA). Given the exploratory nature of this study 95% CI were used to guide the interpretation of the associations^31^.

### Ethics approval

The protocol was approved by the Ethics and Research Committee of the University of Sao Paulo School of Medicine (Comitê de Ética e Pesquisa da Faculdade de Medicina da Universidade de São Paulo, records no. 1.719.856) and the National Commission for Research Ethics (Comissão Nacional de Ética em Pesquisa [CONEP], records no. 2.014.816).

## Results

The final sample size with complete data consisted of 2610 adolescents [52.2% boys; mean age 14.9 (SD: 0.71)]. The preponderance of the sample had white skin color/ethnicity (44.5%), third tercile of wealth index (34.1%), had mothers who completed secondary education (34.9%) and whose mothers’ employment status was unemployed (37.1%) (Table 1).

**Table 1:** Characteristics of adolescents by total physical activity level (< 60 min/day vs ≥ 60 min/day). SP-PROSO, São Paulo, 2017.

Variable	n (%)	< 60 min/day	≥ 60 min/day
Total	2610	2271 (87.0)	339 (13.0)
**Sex**			
Boys	1.363 (52.2)	1116 (81.9)	247 (18.1)
Girls	1.247 (47.8)	1155 (92.6)	92 (7.4)
**Skin color/ethnicity**			
White	1.161 (44.5)	1001 (86.2)	160 (13.8)
Black	323 (12.4)	289 (89.5)	34 (10.5)
Mixed	955 (36.6)	825 (86.4)	130 (13.6)
Other	171 (6.5)	148 (86.5)	23 (13.5)
**Wealth scores**			
First tercile	862 (33.0)	782 (90.8)	80 (9.2)
Second tercile	859 (32.9)	748 (87.1)	111 (12.9)
Third tercile	889 (34.1)	733 (82.4)	156 (17.6)
Mother’s education level*			
Incomplete elementary	331 (16.5)	285 (86.1)	46 (13.9)
Incomplete secondary	287 (14.3)	256 (89.2)	31 (10.9)
Complete secondary	698 (34.9)	605 (86.7)	93 (13.3)
Complete higher	684 (34.4)	580 (84.8)	104 (15.2)
**Mother’s employment status**			
None	969 (37.1)	904 (93.3)	65 (6.7)
Part-time	769 (29.5)	679 (88.3)	90 (11.7)
Full-time	872 (33.4)	750 (86.0)	122 (14.0)

*The maternal education variable had substantial missing data (n = 2000).

The proportion of adolescents who did ≥ 60 min/day of total physical activity was 13%, with a higher proportion in boys (18.1%) than girls (7.4%); those in the third tercile of wealth score (17.6%) than in the first tercile of wealth score (9.2%); those whose mothers completed higher education (15.2%) than those whose mothers did not complete middle school (10.9%); and those whose mothers had a full time job (14.0%) than those whose mothers were unemployed (6.7%) (Table 1).

Most of the schools were municipal public (40.0%) and had at least two sports courts (53.0%); 92% of schools did not have swimming pools in usable condition, 74% did not have locker rooms in usable condition (73.9%) and 98% did not have running/athletics tracks (98.3%). Over half schools had accessible entrances for student cyclists, pedestrian crossings, and two or more physical activity facilities. Over 50% of schools did not have bike racks and speed limit signs around the school (Table 2).

**Table 2:** Characteristics, physical activity facilities and access to school environment in the selected schools. SP-PROSO, São Paulo, 2017.

Variable	n (%)
**Type of school**	119
Municipal public	48 (40.0)
State public	38 (32.2)
Private	33 (27.8)
**Physical activity facilities in the school**	
Sports courts	
≤ 1	42 (36.5)
2	61 (53.0)
≥ 3	12 (10.5)
Swimming pool	
None and not fit for use	106 (92.2)
In usable conditions	9 (7.8)
Locker room	
None and not fit for use	85 (73.9)
In usable conditions	30 (26.1)
Running/athletics tracks	
No	113 (98.3)
Yes	2 (1.7)
**Access to school related to physical activity**	
Accessible entrance for student cyclists	
No	45 (39.1)
Yes	70 (60.9)
Bike racks	
No	82 (71.3)
Yes	33 (28.7)
Speed limit signal around the school	
No	75 (65.2)
Yes	40 (34.8)
Pedestrian crossings	
No	51 (44.3)
Yes	64 (55.6)
Number of physical activity facilities	
≤ 1	24 (20.9)
2–4	48 (41.7)
≥ 5	43 (37.4)

In the multivariable model, three or more (OR: 1.62; 95% CI: 1.15; 2.30) sports courts, swimming pool available in usable conditions (OR: 1.45; 95% CI: 1.01; 2.10), running/athletics tracks (OR: 2.35; 95% CI: 1.07; 5.18), and have bike racks (OR: 1.38; 95% CI: 1.07; 1.78) were positively associated with higher odds of the achievement of a total minimum recommended physical activity (here defined as ≥ 60 min/day) (Table 3).

**Table 3:** Association between school environment and total physical activity in adolescents.

Variable	Total physical activity (≥ 60 min/day)					
	Prevalence		Unadjusted model*		Adjusted model**	
	%	95% CI	OR	95% CI	OR	95% CI
**Sports court**						
≤ 1	12.1	10.1; 14.1	Ref.		Ref.	
2	12.3	10.6; 14.1	1.02	0.79; 1.31	1.01	0.78; 1.31
≥ 3	17.7	14.0; 22.1	**1.56**	**1.11; 2.18**	**1.62**	**1.15; 2.30**
**Swimming pool**						
None and not fit for use	12.5	11.2; 13.9	Ref.		Ref.	
In usable conditions	16.9	12.7; 21.5	**1.42**	**1.01; 2.01**	**1.45**	**1.01; 2.10**
**Locker room**						
None and not fit for use	12.1	10.0; 14.1	Ref.		Ref.	
In usable conditions	14.5	12.0; 17.0	1.18	0.89; 1.55	1.15	0.86; 1.53
**Running/athletics tracks**						
No	12.8	11.6; 14.1	Ref.		Ref.	
Yes	25.0	11.1; 38.9	**2.27**	**1.06; 4.87**	**2.35**	**1.07; 5.18**
**Accessible entrance for student cyclists**						
No	12.9	10.9; 15.3	Ref.		Ref.	
Yes	13.1	11.2; 14.5	1.02	0.81; 1.28	1.04	0.82; 1.32
**Bike racks**						
No	11.7	10.2; 13.1	Ref.		Ref.	
Yes	15.7	13.2; 18.3	**1.40**	**1.11; 1.77**	**1.38**	**1.07; 1.78**
**Speed limit signs around the school**						
No	12.6	10.9; 14.1	Ref.		Ref.	
Yes	13.8	11.5; 15.8	1.11	0.87; 1.41	1.12	0.85; 1.46
**Pedestrian crossings**						
No	12.3	10.4; 14.2	Ref.		Ref.	
Yes	13.5	11.7; 15.3	1.13	0.88; 1.40	1.13	0.89; 1.45
**Number of physical activity facilities**						
≤ 1	6.8	3.1; 13.7	Ref.		Ref.	
2–4	13.2	10.8; 15.4	2.06	0.81; 5.22	2.03	0.79; 5.20
≥ 5	13.1	11.5; 14.7	2.04	0.82; 5.14	2.01	0.79; 5.08

Values in bold indicate significant associations; *95% CI* 95% confidence intervals, *OR* odds ratio.

*Number of adolescents enrolled in the school as first level and type of school as second level.

**Number of adolescents enrolled in the school as first level, type of school as second level, adjusted by sex, age (years), skin color/ethnicity, and mother’s employment status.

The presence of two (OR: 1.28; 95% CI: 1.07; 1.52) and three or more (OR: 1.34; 95% CI: 1.03; 1.73) sports courts, swimming pool available in usable conditions (OR: 1.42; 95% CI: 1.21; 1.67), speed limit signals around the school (OR: 1.29; 95% CI: 1.09; 1.53), and pedestrian crossings (OR: 1.34; 95% CI: 1.14; 1.58) positively associated with higher odds of the physical education classes (here defined as ≥ 2 week) (Table 4).

**Table 4:** Association between school characteristics and participation in physical education classes in adolescents.

Variable	Physical education classes (≥ 2 week)					
	Prevalence		Unadjusted model*		Adjusted model**	
	%	95% CI	OR	95% CI	OR	95% CI
**Sports court**						
≤ 1	46.7	43.4; 49.9	Ref.		Ref.	
2	53.0	50.3; 55.9	**1.28**	**1.08; 1.52**	**1.28**	**1.07; 1.52**
≥ 3	54.2	48.8; 59.6	**1.35**	**1.05; 1.73**	**1.34**	**1.03; 1.73**
**Swimming pool**						
None and not fit for use	49.5	47.5; 51.7	Ref.		Ref.	
In usable conditions	63.5	57.5; 69.0	**1.77**	**1.35; 2.32**	**1.42**	**1.21; 1.67**
**Locker room**						
None and not fit for use	52.2	49.0; 55.4	Ref.		Ref.	
In usable conditions	50.5	47.1; 54.4	0.89	0.81; 1.19	0.97	0.80; 1.18
**Running/athletics tracks**						
No	50.7	48.8; 52.8	Ref.		Ref.	
Yes	62.5	43.8; 78.1	1.61	0.78; 3.32	1.64	0.80; 3.39
**Accessible entrance for student cyclists**						
No	48.6	45.4; 54.6	Ref.		Ref.	
Yes	52.4	49.7; 55.0	1.16	0.99; 1.36	1.17	0.99; 1.37
**Bike racks**						
No	49.4	46.9; 51.7	Ref.		Ref.	
Yes	54.0	50.3; 57.4	**1.20**	**1.01; 1.42**	1.16	0.98; 1.38
**Speed limit signs around the school**						
No	48.1	45.7; 50.5	Ref.		Ref.	
Yes	54.5	51.2; 57.5	**1.29**	**1.09; 1.52**	**1.29**	**1.09; 1.53**
**Pedestrian crossings**						
No	46.6	43.6; 49.3	Ref.		Ref.	
Yes	54.0	51.5; 56.8	**1.34**	**1.14; 1.57**	**1.34**	**1.14; 1.58**
**Number of physical activity facilities**						
≤ 1	46.4	34.8; 58.0	Ref.		Ref.	
2–4	49.2	45.9; 52.7	1.12	0.68; 1.83	1.21	0.73; 2.01
≥ 5	51.9	49.6; 54.3	1.24	0.77; 2.02	1.33	0.81; 2.18

Values in bold indicate significant associations; *95% CI* 95% confidence intervals, *OR* odds ratio.

*Number of adolescents enrolled in the school as first level and type of school as second level.

**Number of adolescents enrolled in the school as first level, type of school as second level, adjusted by sex, age (years), skin color/ethnicity, and mother’s employment status.

We did not observe associations between any physical activity facilities and extracurricular sports activities in schools with leisure-time physical activity (here defined as ≥ 60 min/day) (Table 5).

**Table 5:** Association between school characteristics and leisure time physical activity in adolescents.

Variable	Leisure time physical activity (≥ 60 min/day)					
	Prevalence		Unadjusted model*		Adjusted model**	
	%	95% CI	OR	95% CI	OR	95% CI
**Sports court**						
≤ 1	9.0	6.6; 11.4	Ref.		Ref.	
2	8.5	6.5; 10.4	0.93	0.64; 1.36	0.96	0.66; 1.41
≥ 3	7.7	4.5; 11.3	0.84	0.47; 1.48	0.90	0.51; 1.61
**Swimming pool**						
None and not fit for use	8.5	7.1; 9.9	Ref.		Ref.	
In usable conditions	9.8	5.5; 14.7	1.17	0.68; 2.02	1.26	0.72; 2.19
**Locker room**						
None and not fit for use	10.0	7.9; 12.8	Ref.		Ref.	
In usable conditions	7.1	4.9; 9.4	0.82	0.52; 1.28	0.83	0.52; 1.31
**Running/athletics tracks**						
No	8.6	7.2; 10.0	Ref.		Ref.	
Yes	9.5	3.9; 15.1	1.12	0.25; 4.90	1.11	0.25; 4.88
**Accessible entrance for student cyclists**						
No	8.1	6.4; 9.9	Ref.		Ref.	
Yes	9.3	7.1; 11.6	1.15	0.81; 1.64	1.14	0.80; 1.63
**Bike racks**						
No	7.8	6.0; 9.5	Ref.		Ref.	
Yes	10.2	7.8; 12.7	1.34	0.94; 1.92	1.35	0.94; 1.94
**Speed limit signs around the school**						
No	9.3	7.5; 11.2	Ref.		Ref.	
Yes	7.5	5.6; 9.9	0.78	0.53; 1.15	0.79	0.54; 1.17
**Pedestrian crossings**						
No	8.5	6.4; 10.6	Ref.		Ref.	
Yes	8.7	6.8; 10.7	1.01	0.71; 1.45	1.06	0.74; 1.52
**Number of physical activity facilities**						
≤ 1	4.3	0.8; 7.8	Ref.		Ref.	
2–4	8.8	6.5; 11.3	2.17	0.51; 9.23	2.11	0.49; 9.03
≥ 5	8.7	7.0; 10.6	2.13	0.51; 8.95	2.15	0.51; 9.06

*95% CI* 95% confidence intervals, *OR* odds ratio.

*Number of adolescents enrolled in the school as first level and type of school as second level.

**Number of adolescents enrolled in the school as first level, type of school as second level, adjusted by sex, age (years), skin color/ethnicity, and mother’s employment status.

The bike racks (OR: 1.27; 95% CI: 1.07; 1.51), speed limit signs around the school (OR: 1.42; 95% CI: 1.19; 1.68), and pedestrian crossings (OR: 1.31; 95% CI: 1.11; 1.54) were positively associated with higher odds of the active commuting (here defined as ≥ 1 week) (Table 6).

**Table 6:** Association between school characteristics and active commuting to/from school in adolescents.

Variable	Active commuting (≥ 1 week)					
	Prevalence		Unadjusted model*		Adjusted model**	
	%	95% CI	OR	95% CI	OR	95% CI
**Sports court**						
≤ 1	62.8	59.7; 65.9	Ref.		Ref.	
2	58.6	55.9; 61.4	0.84	0.64; 1.04	0.81	0.60; 1.02
≥ 3	55.2	50.1; 60.9	0.73	0.40; 1.03	0.72	0.42; 1.03
**Swimming pool**						
None and not fit for use	60.2	58.3; 62.3	Ref.		Ref.	
In usable conditions	54.5	48.6; 60.5	0.79	0.61; 1.02	0.79	0.60; 1.03
**Locker room**						
None and not fit for use	58.9	55.4; 62.1	Ref.		Ref.	
In usable conditions	56.6	53.4; 59.5	0.84	0.69; 1.01	0.85	0.70; 1.04
**Running/athletics tracks**						
No	59.7	57.8; 61.6	Ref.		Ref.	
Yes	60.6	42.4; 75.8	1.04	0.51; 2.10	1.04	0.51; 2.11
**Accessible entrance for student cyclists**						
No	58.9	55.9; 61.8	Ref.		Ref.	
Yes	60.2	57.7; 62.7	1.05	0.89; 1.23	1.06	0.89; 1.25
**Bike racks**						
No	56.2	52.7; 59.9	Ref.		Ref.	
Yes	61.3	59.2; 63.6	**1.23**	**1.05; 1.45**	**1.27**	**1.07; 1.51**
**Speed limit signs around the school**						
No	54.7	51.5; 58.0	Ref.		Ref.	
Yes	62.4	60.0; 65.0	**1.37**	**1.16; 1.62**	**1.42**	**1.19; 1.68**
**Pedestrian crossings**						
No	57.2	54.7; 60.0	Ref.		Ref.	
Yes	63.0	60.1; 66.1	**1.27**	**1.08; 1.49**	**1.31**	**1.11; 1.54**
**Number of physical activity facilities**						
≤ 1	64.3	52.9; 75.7	Ref.		Ref.	
2–4	61.5	58.2; 64.8	0.88	0.53; 1.47	0.84	0.50; 1.42
≥ 5	58.5	56.1; 60.6	0.78	0.47; 1.29	0.72	0.43; 1.21

Values in bold indicate significant associations; *95% CI* 95% confidence intervals, *OR* odds ratio.

*Number of adolescents enrolled in the school as first level and type of school as second level.

**Number of adolescents enrolled in the school as first level, type of school as second level, adjusted by sex, age (years), skin color/ethnicity, and mother’s employment status.

## Discussion

This cross-sectional study examined the associations of school environment with total physical activity and domain-specific physical activity in adolescents from the largest city in Latin America, Sao Paulo. We found that higher number of sports courts (≥ 3), swimming pool available in usable conditions, running/athletics tracks, and bike racks were positively associated with total physical activity (≥ 60 min/day). We found specific associations between school characteristics and domains of physical activity in adolescents. Our study provides highlight the importance of school environment to promote physical activity for adolescents.

We found a positive association between the presence of two or more sports courts, swimming pools available in usable conditions, speed limit signs around the school, and pedestrian crossings and participation in physical education classes. Previous studies have shown that adolescents spent more time in physical education classes in schools that had sports court availability^21,32^. A systematic review concluded that students attending schools with the higher number of physical environment features may have increased physical activity (compared to lower number of facilities)^33^. Therefore, improving the amount, variety and condition of the facilities in the school environment may be relevant strategies to increase physical activity at school and enhance the participation in physical education classes. The present results corroborate previous studies that have attributed a significant association of the school context variability on physical activity. It is likely that, in the case of the present study, this fact was due to the sampling randomness in each school, as well as the possible similarity regarding the infrastructure.

We not found significant associations between any physical activity facilities and extracurricular sports activities in schools with leisure time physical activity (≥ 60 min/day). On the other hand, Rezende et al.^21^ used data collected for PeNSE (Pesquisa Nacional de Saúde do Escolar—National Survey of School Health) showed positive associations between number of sports court (≥ 2), the presence of running/athletics track, and swimming pool available in usable condition with leisure-time physical activity in adolescents from Brazil. The difference in these findings may be due to the different cut-offs. The authors concluded that school context has important potential to increase the engagement of adolescents in physical activity. Therefore, school environment has a fundamental role in allowing adolescents, who spend part of their day in school, to have a more active lifestyle^21^. Furthermore, there is still no physical activity guidelines for each domain of physical activity^1^. Of note, leisure time physical activity contribute to 41.3% of total physical activity in adolescents from Sao Paulo^9^. Therefore, physical activity interventions, especially recreational activities, are important to increase the total physical activity in adolescent from Sao Paulo.

Our study also showed an association between school environment (bike racks, speed limit signs around the school, and pedestrian crossings) and active commuting (≥ 1 week). Active commuting is an essential source of physical activity and has been associated with higher physical activity levels in adolescents^9^. Safety, social support for commuting and the built environment have been reported to be important determinants of active commuting in adolescents from high-income countries^14^. Furthermore, density of exercise facilities and urbanization (i.e., urban versus rural residences) are positively associated with physical activity^13^. Distance from home to school is the most common barrier to active commuting to school, especially because it is the primary factor in the parents’ decision-making process for or against allowing their child to walk or cycle to school^34^. In addition, it may not be feasible to walk or cycle to school if the distance is too far in medium-sized towns possibly because of higher traffic density^35^. Sao Paulo is among the 10 most urbanized, highly disorganized cities, with heavy traffic, air and noise pollution, rising crime rates and high-income inequality^8^. Future research should address the physical and social environmental determinants of active commuting such as safety, presence of sidewalks and bicycle lanes, and availability of infrastructure to verify this hypothesis in adolescents from low-income to middle income countries.

Our results are also consistent with previous studies showing a positive association between the amount of school facilities and adolescent physical activity^36,37^. A significant number of physical activity facilities must be implemented in schools, as this might help to offer a diversity of opportunities for physical activity^38^. Possibly, more recreational activities and spaces with equipment and/or ‘obstacles (natural or built), which stimulate the increase of the total physical activity levels^39^. The proposal for a diverse infrastructure and open school would enhance the capability of the school to promote societal knowledge related to body culture and understanding of concepts such as leisure goals, expression of feelings, affections and emotions, and opportunities to promote, restore and maintain health, one of the aims of the *Programa Saúde na Escola* (Health in Schools Program), in Brazil^40^.

Our study included a large representative sample of adolescents from the city of Sao Paulo to examine the association between school environment and physical activity. However, our findings should be interpreted in the light of some limitations. First, the cross-sectional associations must be interpreted cautiously, as they may not be appropriate for causal inference. Second, *SP-PROSO* study did not include adolescents of all ages and the results may not applicable to all ages. Third, physical activity information was collected through a self-reported questionnaire and therefore measurement error probably occurred. However, this physical activity questionnaire has shown a high relative^28^. The variable of mothers’ education level has considerable missing data. Adolescents with missing maternal education data showed similar physical activity pattern^9^.

## Conclusions

We found that schools with a higher number of physical activity facilities had higher odds of adolescents reaching the physical activity guidelines. Physical activity facilities were differentially associated with domain-specific physical activity, which can be useful to inform the development of future physical activity programs. The school environment may have an important role in allowing adolescents, who spend part of their day in school, to have a more active lifestyle. Prospective studies of school characteristics and physical activity are needed, as well as evidence from intervention studies in order to advance our understanding of these relationships and better inform educational and public health policy, programs and decision-makers.

## Data Availability

Data are available upon reasonable request from the corresponding author.
